# Evaluating AR1001 as Monotherapy from a Phase 2 Study in Mild to Moderate Alzheimer’s Disease Patients

**DOI:** 10.1002/alz70859_107781

**Published:** 2025-12-25

**Authors:** Fred Kim, Tianyang Xi, Han Joo Lee, James Arthur Rock, SangYun Kim, Jai Jun Choung

**Affiliations:** ^1^ AriBio Co., Ltd., San Diego, CA USA; ^2^ AriBio Co., Ltd., Seongnam‐si, Gyeonggi‐do Korea, Republic of (South); ^3^ AriBio Co., Ltd, San Diego, CA USA; ^4^ Seoul National University College of Medicine & Seoul National University Bundang Hospital, Sungnam Korea, Republic of (South)

## Abstract

**Background:**

AR1001 (mirodenafil), a second‐generation oral phosphodiesterase 5 (PDE5) inhibitor, is being investigated as a disease‐modifying therapy for Alzheimer’s disease (AD). The phase 2 trial (AR1001‐ADP2‐US01) aimed to evaluate the safety and efficacy of AR1001 in patients with mild to moderate AD. After 26 weeks of once‐daily oral dosing, 10 mg and 30 mg AR1001 demonstrated acceptable safety profiles in this population. While there were no significant differences between the treatment groups for the primary endpoint, Alzheimer’s Disease Assessment Scale, cognitive subscale 13 (ADAS‐Cog‐13) at Week 26, plasma ptau‐181 and ptau‐217 AD biomarkers were significantly reduced in the 30 mg group compared to placebo.

**Method:**

In this double‐blind, randomized, placebo‐controlled, parallel‐group trial, 210 patients diagnosed with mild to moderate AD were randomized to receive either placebo, AR1001 10 mg, or AR1001 30 mg. Participants were administered treatment once‐daily for 26 weeks. Participants were diagnosed clinically based on 2011 National Institute of Aging and Alzheimer’s Associations criteria and were allowed to be on concomitant AD medication such as acetylcholinesterase inhibitors and NMDA receptor antagonists with at least 3 months of stable dose prior to screening. Pre‐specified subgroup analyses based on concomitant AD medication were conducted for ADAS‐Cog 13 and key plasma biomarkers, including ptau‐181 and ptau‐217.

**Result:**

Sixty‐seven and 69 participants were assigned to the placebo and AR1001 30 mg groups, respectively. At baseline, 36 of 67 (51.4%) participants on placebo and 46 of 69 (65.7%) participants on 30 mg AR1001 were on concomitant AD medication. Participants without concomitant AD medication treated with AR1001 30 mg (monotherapy) demonstrated a statistically significant improvement of 4.019 points over baseline at Week 26 (p=0.012) on ADAS‐Cog 13. AR1001 30 mg monotherapy group also demonstrated reductions of 1.361 pg/ml in plasma ptau‐181 (p=0.023) and 0.426 pg/mL in plasma ptau‐217 compared to placebo at Week 26.

**Conclusion:**

The subgroup analysis of participants on AR1001 30 mg without concomitant AD medication suggests potential for AR1001 as a monotherapy for the treatment of Alzheimer’s disease.

